# Relevance of Pressure Recovery in a Young Patient With Aortic Stenosis and Small-Caliber Aorta

**DOI:** 10.1016/j.jaccas.2024.103071

**Published:** 2025-03-05

**Authors:** Jan-Christian Reil, Hazem Omran, Nael Hasan, Gert-Hinrich Reil, Lech Paluszkiewicz

**Affiliations:** a Klinik für allgemeine und interventionelle Kardiologie, Herz-Diabetes-Zentrum Nordrhein-Westphalen, Bad Oeynhausen, Germany; b Klinik für Thorax- und Kardiovaskularchirurgie, Herz-Diabetes-Zentrum Nordrhein-Westphalen, Bad Oeynhausen, Germany; c Universitätsklinik für Innere Medizin–Kardiologie, Klinikum Oldenburg, Oldenburg, Germany

**Keywords:** aortic valve, echocardiography, hemodynamics

## Abstract

The case concerns a 20-year-old patient with Canadian Cardiovascular Society class II angina who was initially referred for aortic valve replacement because of a suspected high-grade aortic valve stenosis with increased transvalvular gradients (max/mean: 70/40 mm Hg) measured by Doppler echocardiography. Examinations using transesophageal echocardiography and computed tomography showed a sufficiently opening bicuspid aortic valve, excluded supra- and subvalvular stenoses, and measured a narrow aorta (diameter: 2 cm). The explanation for the highly increased gradients across the aortic valve was the pressure recovery (PR) phenomenon, which cannot be detected by Doppler gradients. Distal to a stenosis kinetic energy is converted back into potential energy, most effectively in small aortas (area: <3 cm^2^). This reduces the actual transvalvular pressure gradient, which can directly be determined with cardiac catheterization. Accordingly, invasive measurements showed a moderate aortic stenosis (mean transvalvular pressure: 19 mm Hg), almost identical to the PR-corrected Doppler measurements. A high-grade stenosis of the proximal left anterior descending artery was treated interventionally, which could explain the angina symptoms.

## Graphical Abstract

**Figure:**
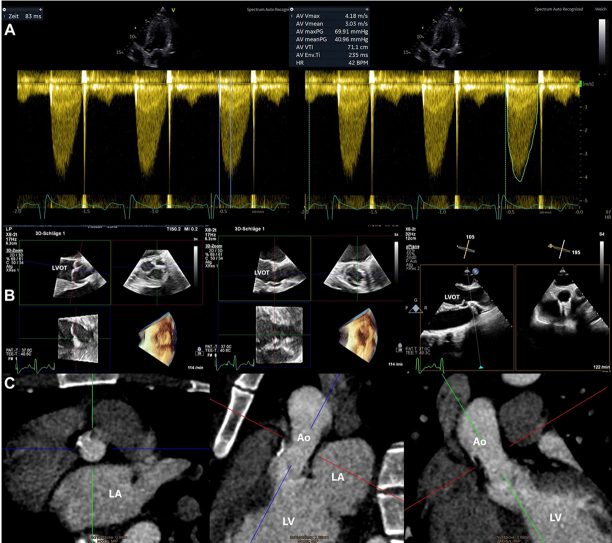

## History of Presentation

A 22-year-old patient was admitted for surgery because of a suspected severe aortic valve stenosis in our hospital. He suffered from angina pectoris complaints of Canadian Cardiovascular Society class II. The external echocardiography showed a mean gradient of approximately 40 mm Hg across the aortic valve with a valve opening area of approximately 0.8 cm^2^ without aortic regurgitation. There were also no other pre-existing illnesses.

**Take-Home Messages**

• Pressure recovery of aortic stenosis behind the aortic root depends on the ratio of the valve opening area (effective orifice area) and the cross-sectional area of the aorta (A_A_) and can reach a maximum of 50%, which is called relative pressure recovery.
• The pressure recovery cannot be measured with “standard” Doppler echocardiography.
• In the case of narrow aortas (A_A_ <3 cm^2^) or conflicting findings, the pressure gradient across the aortic valve should be measured invasively or a Doppler-based calculation including correction for pressure recovery should be performed.

**Figure 1:**
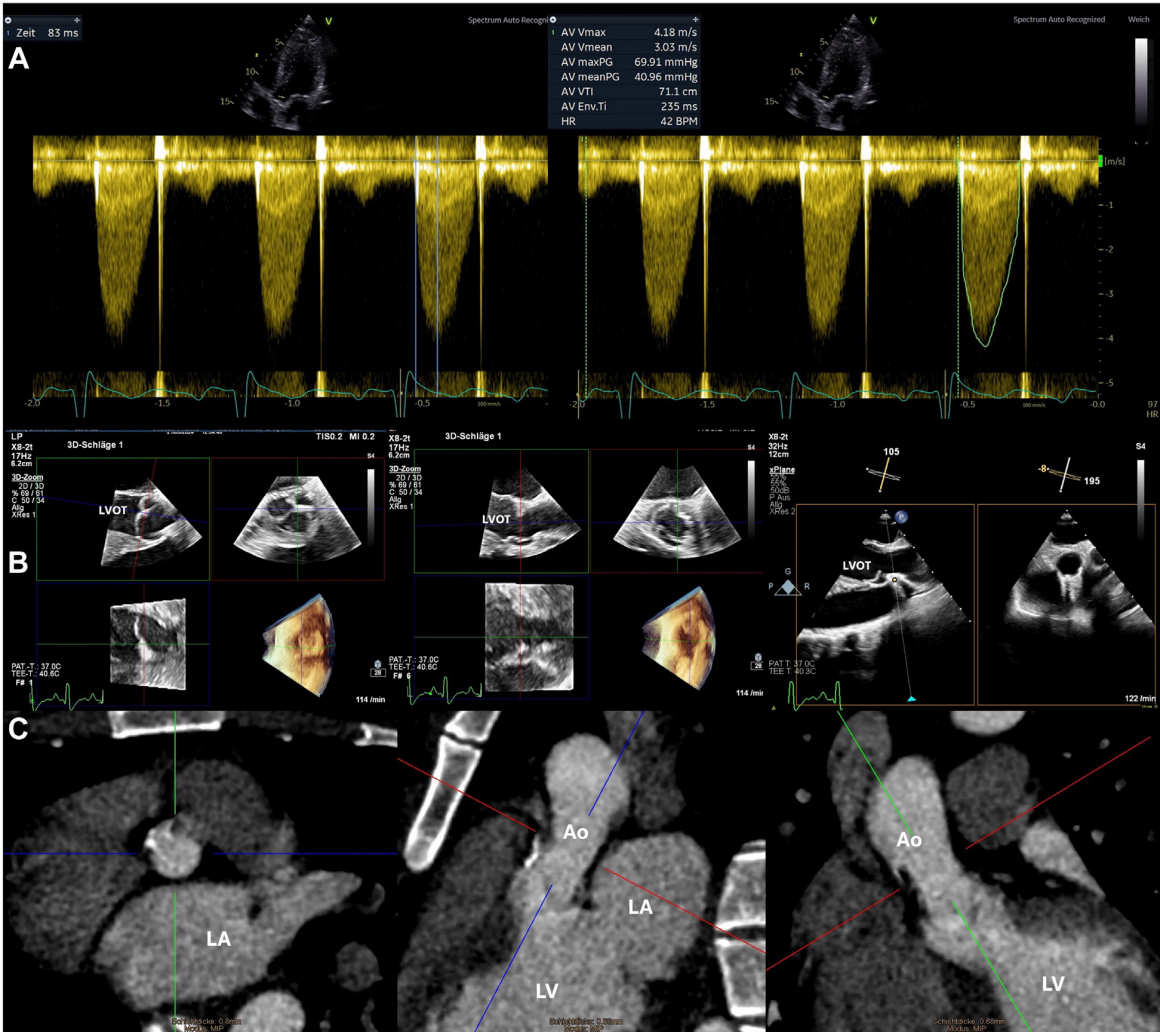
Echocardiographic and Computed Tomographic Images (A) Echocardiography-derived pressure gradients across the aortic valve including acceleration time. (B and C) Transesophageal echocardiography and computed tomography of the aortic valve and ascending aorta. Ao = aorta; LA = left atrium; LV = left ventricle; LVOT = left ventricular outflow tract.

A preoperative echocardiography revealed a functioning, nonsclerosed bicuspidal aortic valve, despite significant pressure differences (high transvalvular gradient of maximal transvalvular pressure [P_max_]/mean transvalvular pressure [P_mean_], 70/40 mm Hg), an acceleration time of 83 ms, and a cardiac output of 6.5 L/min (Figure 1A). On auscultation, only a soft systolic midpeaking murmur was audible over the second intercostal space on the left parasternal side without radiation into the carotids, which would also negate a high-grade aortic valve stenosis. These conflicting results led to a postponement of the surgery for additional tests.

## Differential Diagnosis

In addition to aortic valve stenosis, increased pressure gradients across the aortic valve can also be caused by a subvalvular membrane, dynamic obstruction of the outflow tract as in hypertrophic obstructive cardiomyopathy, or supra-aortic stenosis. Furthermore, diseases with significantly increased cardiac output, such as hyperthyroidism, can produce increased gradients. Alternatively, Doppler gradients may overestimate the true gradient across the aortic valve by neglecting pressure recovery (PR) behind the aortic root.

## Investigations

### Case-based calculation of PR

Subsequent transesophageal echocardiography showed the aortic valve was opening well, indicating only mild stenosis (opening area of 1.5 cm^2^) (Figure 1B), and ruled out other potential obstructions like supra- and subvalvular stenoses and hypertrophic obstructive cardiomyopathy. A conspicuous narrowing of the ascending aorta with a diameter of approximately 2 cm was observed, confirmed by computed tomography (Figure 1C). Thyroid laboratory values were unremarkable, and thus hyperthyroidism was ruled out.

**Figure 2:**
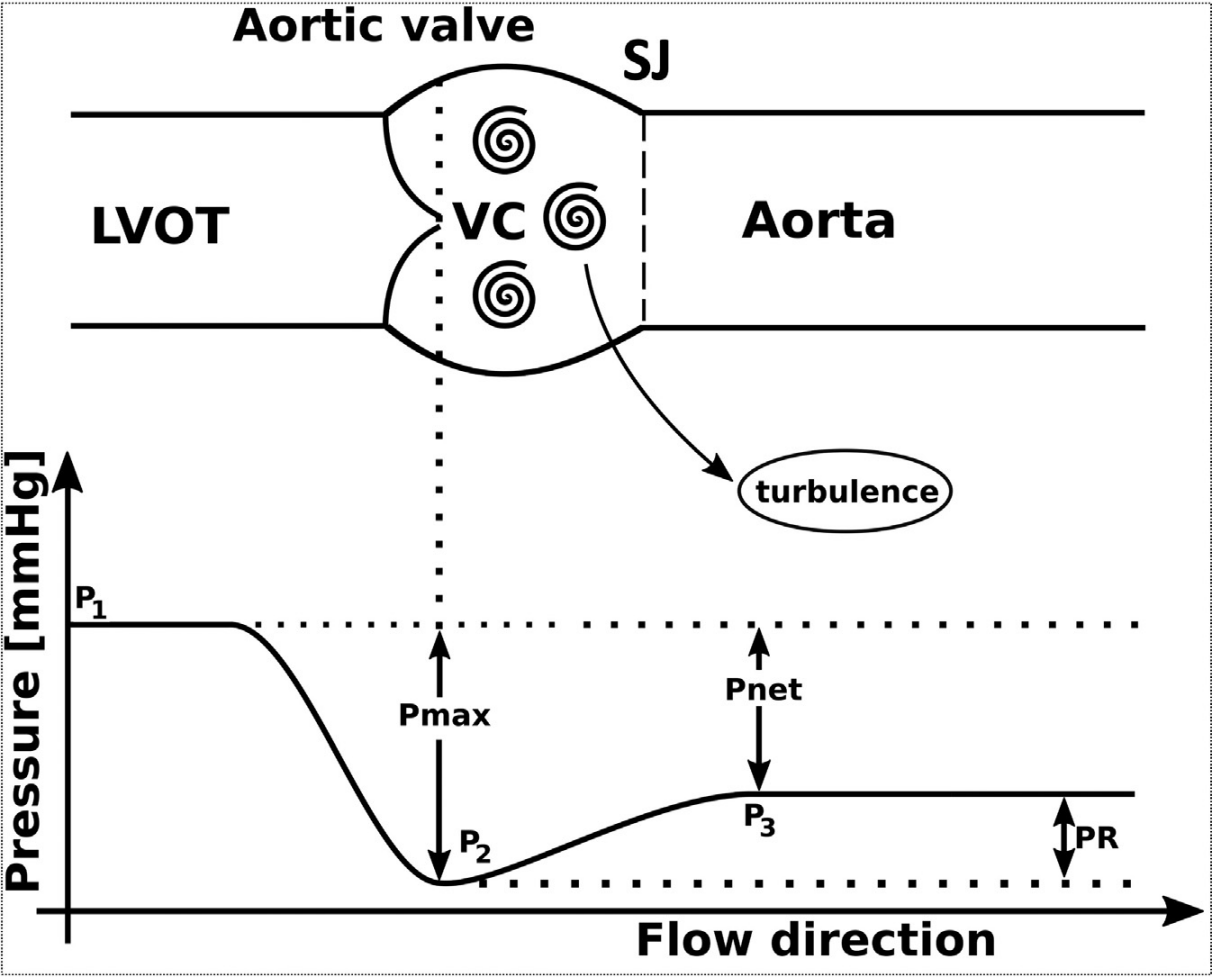
Background of Pressure Recovery Theoretical background of pressure recovery (PR) and definition of different pressure values (P1-P3) in the left ventricle, VC, and behind the sinutubular junction (SF). Distal to the VC (P_max_ = P1-P2), accelerated blood flow of aortic stenosis slows down and turbulence leads to energy loss by converting kinetic energy into heat. In addition, a smaller part of the remaining kinetic energy behind the SJ is reconverted into potential energy (P3), which explains the PR phenomenon. Invasive measurements by heart catheterization consider PR by measuring P_net_. Echocardiography-based Doppler measures P_max_ and ignores PR. LVOT = left ventricular outflow tract; P_max_ = maximal transvalvular pressure; P_net_ = pressure difference; VC = vena contracta.

Echocardiography captures the peak pressure gradient at the narrowest point behind the valve (ie, between the left ventricle and the aortic valve at the vena contracta) but misses the PR phase during which kinetic energy is converted back in the adjacent ascending aorta into potential energy, leading to an overestimation of the pressure difference^1^, ^2^, ^3^, ^4^, ^5^ (Figure 2). PR is determined by the size of the valve opening area (effective orifice area [EOA], calculated as 1.5 cm^2^) in relation to the aorta’s cross-sectional area (A_A_) measured adjacent to the sinutubular junction^4^ and is favored by narrower vessels (A_A_ <3.14 cm^2^).^5^^,^^6^ The shape of the valve opening area (round, elliptical, or slotted) or the design of the valve (tricuspid, bicuspid, or inicuspid) does not affect PR. By calculating the PR, validated by invasive methods, we obtained a more accurate pressure gradient across the aortic valve.^2^ Specifically, applying the formula for PR in relation to the maximum pressure (P_max_) resulted in a value of 0.48 (PR index [PRI] or relative PR) close to the theoretical maximum of 0.50.^6^ This calculation led to an adjusted “actual” pressure difference (P_net_) of 34 mm Hg, significantly lower than initially assumed.

### Case-based step-by-step calculation of PR values for clinical practice

Using the echocardiography parameters, aortic diameter (D), EOA of the aortic valve, and P_max_, we calculated the following formulas:

$$A_{A}=\left(D^{2}/4\right)\times \pi =\left(4/4\right)\times 3.14=3.14\;{\text{cm}}^{2}\;\left(D=2\;\text{cm}\right)$$

$$\text{Relative}\;\mathrm{PR}=\mathrm{PR}/P_{\max}=\text{PRI}$$

$$PRI=2\times \left(EOA/A_{A\;}–\;{\left(EOA/A_{A}\right)}^{2}\right)\left(EOA=1.5\;{cm}^{2},A_{A}=3.1\;{cm}^{2}\right)$$

$$PRI=2\times \left(1.5/3.1\right)\;–\;{\left(1.5/3.1\right)}^{2})=0.48$$

We used the following formulas to correct maximal values (Figure 2):

$${\mathrm{PR}}_{\max}=P_{\max}\times \text{PRI}=68\;\text{mm}\;\text{Hg}\times 0.48=34\;\text{mm}\;\text{Hg}$$

$$P_{\text{net}}=P_{\max}–\;\mathrm{PR}=68\;–\;34\;\text{mm}\;\text{Hg}=34\;\text{mm}\;\text{Hg}$$

Finally, we used the following formulas to correct mean values:

$${\mathrm{PR}}_{\text{mean}}=P_{\text{mean}}\times \text{PRI}=40\;\text{mm}\;\text{Hg}\times 0.48=19.2\;\text{mm}\;\text{Hg}$$

$$P_{\text{net}}=P_{\text{mean}}\;–\;{\mathrm{PR}}_{\text{mean}}=40\;–\;19.2=20.8\;\text{mm}\;\text{Hg}$$

## Management

**Figure 3:**
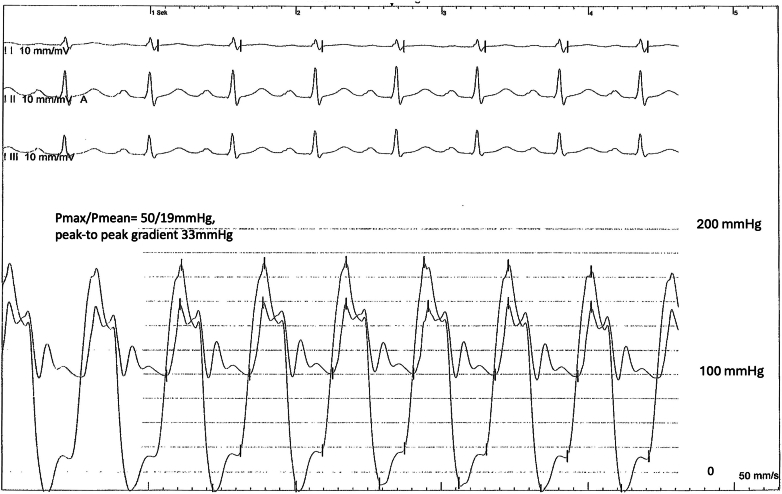
Invasive Hemodynamics Simultaneous invasive pressure traces of the left ventricle and aorta as well as electrocardiographic recordings. P_max_/P_mean_ = maximal transvalvular pressure/mean transvalvular pressure.

Additionally, an invasive aortic valve pressure measurement was performed to verify the unexplained increased pressure gradient and to clarify that PR might play an important role in the patient. The pressure measurements between the left ventricle and ascending aorta showed a transvalvular gradient of P_mean_ of 19 mm Hg and a P_max_ of 50 mm Hg, confirming a mild to moderate aortic stenosis (Figure 3). The invasively measured P_mean_ of 19 mm Hg is almost identical to the PR-corrected P_mean_ value of the Doppler signal and confirmed the correction procedure. Compared with the P_mean_, the P_max_ value was too high and may be explained at least in part by the catheter’s sling motion under the flow conditions. At the same session, a proximal high-grade left anterior descending artery stenosis was diagnosed, which was treated interventionally and can be considered the cause of the angina symptoms.

## Discussion

The present case highlights the difficulty of relying solely on Doppler gradients from echocardiography to diagnose aortic valve stenosis. Without considering PR, there is a risk of overestimating the severity of stenosis. This risk can be reduced by using invasive measurements or considering invasively confirmed Doppler-based calculations taking PR into account.

At a time when echocardiographically obtained Doppler gradients are the most important diagnostic method for quantifying aortic stenosis and invasive quantitative measurements are rarely performed, this case should once again draw attention to the problem of PR in aortic stenosis, which has been well known for decades but is largely forgotten in everyday clinical practice. In the case of conflicting findings, such as a narrow aorta (<3 cm^2^) and borderline valve opening areas of 0.8 to 1.0 cm^2^, a Doppler-calculated PR should be used to correct the valve opening area.^3^^,^^4^

## Conclusions

Pure Doppler measurements may overestimate the transvalvular gradient across the aortic valve, especially in narrow aortic areas (<3 cm^2^), if PR is not taken into account.

## Funding Support and Author Disclosures

Publication of the article was funded by Ruhr University open access funds. The authors have reported that they have no relationships relevant to the contents of this paper to disclose.
